# Transactions—Michigan State Dental Society

**Published:** 1882-11

**Authors:** 


### Transactions.

The volume of transactions of the last annual meeting of the Michigan State Dental Society has recently made its appearance. It contains much of interest, and gives good evidence of the working ability of this, one of our oldest State dental societies. This society, at this meeting, celebrated its twenty-fifth anniversary, a full account of which is given in this volume. A very complete history of dentistry in the State of Michigan from its beginning to the present time is embraced in the proceedings of this meeting. It contains matter of much interest for all, but especially for those of Michigan.

This was collated and prepared by Dr. A. T. Metcalf, of Kalamazoo. The work is published by, and can be obtained of, Mr. F. S. Ackerman, of Detroit.

